# Interplay Among Endothelial Dysfunction, NLRP3 Pathway Activation, and microRNAs in the Pathogenesis of Preeclampsia

**DOI:** 10.3390/diseases14040118

**Published:** 2026-03-26

**Authors:** Daniela Alves Pereira, Priscila Rezeck Nunes, Marcelo Rizzatti Luizon, Valéria Cristina Sandrim

**Affiliations:** 1 Department of Biophysics and Pharmacology, Institute of Biosciences, Universidade Estadual Paulista (UNESP), Botucatu 18618-689, SP, Brazil; da.pereira@unesp.br (D.A.P.); priscila.nunes@unesp.br (P.R.N.); luizonmr@gmail.com (M.R.L.); 2 Department of Genetics, Ecology and Evolution, Institute of Biological Sciences, Federal University of Minas Gerais, Belo Horizonte 31270-901, MG, Brazil

**Keywords:** preeclampsia, endothelial dysfunction, NLRP3, miRNAs, inflammation

## Abstract

Preeclampsia (PE) is a leading cause of maternal and perinatal complications and is classified by early or late onset according to the gestational age. The complex pathogenesis of PE involves placental ischemia, oxidative stress, angiogenic imbalance, and inflammation, all of which contribute to impaired placentation and widespread maternal endothelial dysfunction. These mechanisms drive hypertension, multi-organ involvement, and increased long-term cardiovascular risk. Parallel research highlighted the role of the NLRP3 inflammasome, a multiprotein complex that, upon activation, increases the gene expression, processing, and release of the pro-inflammatory cytokines IL-1β and IL-18. The NLRP3 pathway is markedly upregulated in placentas from pregnant women with PE, where endogenous danger signals stimulate inflammasome activation and amplify inflammation. Increasing evidence indicates that microRNAs (miRNAs) help regulate inflammatory processes, including the NLRP3 inflammasome, thereby affecting placental function and maternal adaptation. Although several immunoregulatory miRNAs may influence NLRP3 activity, their specific contribution to inflammasome regulation in PE remains insufficiently understood. Understanding these interactions could reveal new therapeutic targets for PE. In this narrative review, we explore the interconnected roles of endothelial dysfunction, inflammasome activation, and miRNA-mediated regulation in the pathogenesis of PE.

## 1. Introduction

Preeclampsia (PE) is progressive hypertensive condition of pregnancy that affects multiple organ systems and presents with newly developed hypertension, characterized as systolic blood pressure ≥ 140 mmHg and diastolic blood pressure ≥ 90 mmHg along with proteinuria (i.e., >30 mg/mol protein; creatinine ratio; 300 mg/24 h) or other evidence of maternal organ dysfunction, which may include hepatic impairment, renal insufficiency, pulmonary edema, and neurological or visual symptoms. It typically occurs in the latter half of pregnancy or after delivery [1]. PE is classified as early-onset (before 34 weeks, more severe) or late-onset (after 34 weeks, milder and closer to term). It affects approximately 2–8% of pregnancies and it is the major cause of maternal and perinatal morbidity and mortality, particularly in early-onset cases [2,3]. PE is responsible for an estimated 76,000 maternal deaths and 500,000 infant deaths each year worldwide [3]. PE has a multifactorial pathogenesis driven by genetic, environmental, and maternal factors, as well as metabolic dysfunctions and an impaired antioxidant defense [4,5,6]. It is commonly described by a two-stage disease model [7]. The first stage involves abnormal placentation with defective spiral artery remodeling, which leads to reduced placental perfusion and impaired oxygen and nutrient delivery to the fetus [8]. In the second stage, placental stress promotes the release of antiangiogenic factors and inflammatory mediators into maternal circulation, which triggers systemic inflammation, immune dysregulation, hypercoagulability, and endothelial and platelet dysfunction [9]. Together, these processes result in widespread maternal and fetal organ involvement and hematological complications [7].

Disease progression is driven by immune and inflammatory dysregulation involving both the maternal–fetal interface and the maternal vascular endothelium. Loss of immune tolerance, due to dysfunction of regulatory T cells, uterine natural killer cells, and myeloid-derived suppressor cells, promotes excessive effector and Th17 responses and placental inflammation [10]. This inflammatory environment is reflected by increased circulating biomarkers, including interleukins, TNF-α, C-reactive protein, and adhesion molecules, which may assist in disease stratification and outcome prediction [11].

Accumulating evidence indicates that activation of the NLR Family Pyrin Domain Containing 3 (NLRP3) inflammasome plays a central role in PE pathogenesis. Disruption of immune homeostasis promotes placental inflammation through NLRP3 activation, driven by danger-associated molecular patterns (DAMPs) such as reactive oxygen species (ROS), pro-inflammatory cytokines, ATP, HMGB1 (high-mobility group box-1), and trophoblastic debris released from a hypoxic and necrotic placenta [12]. Hypoxia-related signaling, particularly via Hypoxia-Inducible Factor 1-alpha, HIF-1α, further amplifies inflammation, apoptosis, and thrombosis through enhanced NLRP3 expression [13,14]. In parallel, endothelial cells shift from homeostatic regulators to active inflammatory mediators by sensing danger signals, leading to NLRP3 inflammasome activation and IL-1β production [12,15]. Consistently, increased expression of NLRP3 and its downstream mediators, including caspase-1, IL-1β, and IL-18, has been reported in affected women, underscoring the contribution of endothelial inflammasome activation to disease pathophysiology [16].

Epigenetic mechanisms, including DNA methylation, histone modifications, long non-coding RNAs, and microRNAs (miRNAs), are directly and indirectly involved in the regulation and assembly of the NLRP3 inflammasome [17,18]. Among these, miRNAs control gene expression at the post-transcriptional level by promoting mRNA degradation or translational repression and are essential for normal cellular development and function [19]. NLRP3 activity is tightly regulated by multiple miRNAs through conserved binding sites in its 3′-untranslated region (UTR) of target messenger RNAs (mRNAs) of the NLRP3 complex, highlighting epigenetic modulation as a potential strategy to restore inflammasome homeostasis and prevent tissue damage caused by uncontrolled activation [19].

Several studies also highlight that circulating miRNAs play an important role in the pathophysiology of PE by regulating processes such as trophoblast invasion, angiogenesis, immune responses, and endothelial function. Altered miRNA expression may contribute to disease development and has potential as a minimally invasive biomarker for PE [20].

Although previous reviews have explored the role of the NLRP3 inflammasome in PE, the involvement of microRNAs in PE, or the inflammatory and endothelial mechanisms underlying the disease, these aspects are often discussed separately. However, increasing evidence suggests that microRNAs could regulate key components of inflammatory signaling pathways, including the activation of the NLRP3 inflammasome. Despite the growing recognition of both microRNA dysregulation and NLRP3 activation in the pathophysiology of preeclampsia, studies integrating these two mechanisms remain relatively limited and fragmented. Therefore, this review aims to synthesize current evidence on the regulatory relationship between microRNAs and the NLRP3 inflammasome and to discuss how this interaction may contribute to the inflammatory and endothelial alterations observed in preeclampsia. By focusing on the miRNA–NLRP3 axis, we seek to provide a more integrated perspective on the molecular mechanisms involved in PE and highlight potential avenues for future translational research.

The present narrative review is organized to provide a structured overview of the current knowledge on this topic. We first describe the materials and methods employed in this study. Next, we provide a synthesis of the pathophysiology of PE, with a particular focus on endothelial dysfunction. We then discuss the activation of the NLRP3 inflammasome and its regulatory mechanisms. Subsequently, we explore the role of microRNAs in the regulation of NLRP3 and examine the evidence linking these molecules to PE. Finally, we address the main limitations of the current evidence and outline potential directions for future research.

## 2. Materials and Methods

### 2.1. Study Design

This manuscript was conducted as a structured narrative review aimed at critically synthesizing current evidence on the regulatory interaction between miRNAs and the NLRP3 inflammasome in the context of PE and endothelial dysfunction. Given the substantial heterogeneity in study designs, experimental models, and reported outcomes, a formal systematic review or meta-analysis was not feasible. Therefore, a structured narrative synthesis approach was adopted.

### 2.2. Literature Search Strategy

A literature search was performed in MEDLINE/PubMed to identify relevant studies published for up to ten years. The following search terms and Boolean combinations were used:

• “miRNA AND NLRP3”
• “miRNA AND NLRP3 AND preeclampsia”
• “miRNA AND NLRP3 AND endothelial dysfunction”

Search terms were adapted as necessary to maximize sensitivity. Only articles published in English were considered. The initial search yielded several hundred records. Titles and abstracts were screened for relevance, and potentially eligible articles were subsequently evaluated through full-text review. Studies that fulfilled the inclusion criteria were included in the final synthesis.

### 2.3. Eligibility Criteria

Studies were included if they met the following criteria:

• Original research investigating miRNA regulation of the NLRP3 inflammasome.
• Experimental or clinical studies addressing endothelial dysfunction in PE.
• Mechanistic investigations of inflammasome activation pathways; High-quality review articles providing relevant conceptual or mechanistic insights.

### 2.4. Exclusion Criteria Comprised

• Case reports and conference abstracts without full datasets.
• Studies lacking mechanistic or translational relevance; Articles not directly addressing miRNA–NLRP3 interactions or endothelial dysfunction in PE.

### 2.5. Study Selection and Data Interpretation

Study selection was based on thematic relevance, mechanistic contribution, and scientific robustness. Emphasis was placed on studies with clearly defined methodologies, adequate sample sizes, validated molecular targets, and functional evidence supporting miRNA–inflammasome interactions.

Given the variability across experimental platforms (in vitro models, animal studies, and human clinical samples), quantitative pooling was not attempted. Instead, findings were synthesized narratively and organized into thematic sections to integrate molecular, translational, and clinical perspectives.

### 2.6. Critical Appraisal Approach

To address potential bias inherent in narrative reviews, a critical interpretative framework was applied. The strengths and limitations of key studies were evaluated with attention to:

• Sample size and statistical power.
• Type of experimental model (cellular, animal, human).
• Validation of miRNA–target interactions (e.g., luciferase assays, gain- and loss-of-function studies).
• Consistency of inflammasome activation markers.

Contradictory findings were examined considering methodological differences, including variations in miRNA quantification techniques, normalization strategies, and tissue sources.

### 2.7. Limitations of the Review

This review is limited by reliance on a single database (MEDLINE/PubMed https://pubmed.ncbi.nlm.nih.gov/, Accessed during 1 December 2025 and 31 January 2026) and the absence of a pre-registered systematic protocol, which may introduce selection bias. However, the primary objective was to provide mechanistic integration and conceptual synthesis rather than quantitative aggregation of effect sizes. Efforts were made to ensure transparency in search strategy and study selection to enhance methodological rigor.

## 3. Pathophysiology of Preeclampsia: The Central Role of Endothelial Dysfunction

The pathogenesis of PE is multifactorial and involves a complex interplay of genetic, environmental, and maternal risk factors [3]. PE is characterized by abnormal placentation, impaired maternal cardiovascular adaptation, and inadequate remodeling of the spiral arteries [21]. It is well established that placental ischemia acts as a central event in PE, as the release of factors generated by poor placental perfusion into the maternal circulation promotes systemic endothelial dysfunction [8].

PE and cardiovascular disease (CVD) are closely interconnected conditions, sharing several underlying pathophysiological mechanisms. PE is characterized by endothelial dysfunction, systemic inflammation, oxidative stress, and an imbalance of angiogenic factors, processes also implicated in the development of CVD [22,23]. Growing evidence suggests that the vascular and inflammatory disturbances triggered during PE may persist beyond pregnancy and contribute to long-term cardiovascular alterations. Consequently, women with a history of PE have a significantly increased risk of developing CVD later in life, including chronic hypertension, ischemic heart disease, and stroke. These findings support the concept that PE may not only reflect a preexisting cardiovascular vulnerability but may also actively contribute to the development of cardiovascular disease, highlighting the importance of long-term cardiovascular follow-up in these women [8,24].

These pathophysiological events of PE occur before the clinical onset; however, during pregnancy, an increase in peripheral vascular resistance has been observed, leading to hypertension and proteinuria [25]. Furthermore, this vascular dysfunction leads to platelet aggregation and adhesion, promoting the formation of platelet–leukocyte complexes and resulting in an inflammatory/prothrombotic state [3], conditions that can lead to poor pregnancy outcomes.

Preservation of endothelial barrier integrity is critical for healthy pregnancy progression because the endothelium is physiologically covered by a glycosaminoglycan layer that inhibits thrombin generation and prevents platelet and leukocyte adhesion [26]. The endothelium is strategically located between the blood and the tissues and plays an essential role in integrating and regulating their connection. Important actions of this layer comprehend balancing permeability, coagulation, vascular tone [27], regulation of metabolism and angiogenesis, as well as damage repair [28].

Endothelial barrier dysfunction is characterized by the loss of contact between endothelial cells and the extravasation of plasma, proteins, cells, and solutes [29], and could be a result of any alteration compromising endothelial homeostasis [28]. In preeclamptic pregnancies, the ischemic placenta induces systemic endothelial damage through the release of mediators, causing disruption of vascular homeostasis [30], inflammation and angiogenic imbalance. Systemic vascular dysfunction in PE reflects dysregulated vasoactive mediator production, exaggerated vasoconstrictor responses, impaired endothelial-dependent relaxation, and oxidative stress [31,32]. Endothelial cells respond to the release of mediators by releasing vasoactive molecules that regulate vascular tone through smooth muscle relaxation (EDRFs) or contraction (EDCFs). Dysregulation of these pathways, along with altered smooth muscle sensitivity, likely contributes to the hypertensive phenotype of PE [33].

The impaired endothelium-dependent relaxation observed in pregnancies complicated by PE is partly attributed to reduced bioavailability of nitric oxide (NO) [34] and prostacyclin (PGI_2_) [35]: NO acts via soluble guanylyl cyclase/cGMP–dependent and –independent mechanisms, and PGI_2_ via receptor-mediated cAMP-dependent signaling [36]. In addition, the endothelium produces endothelin (ET)-1, prostanoids, and angiotensin II, which are all vasoconstrictors that have been correlated to vascular dysfunction of hypertensive disorders, including PE [37,38,39].

Furthermore, a profound imbalance in redox homeostasis was observed, contributing to PE pathophysiology, leading to excessive production of ROS and oxidative stress [40]. Increased ROS generation, largely driven by mitochondrial dysfunction, contributes to endothelial damage and impaired vascular function. Mitochondrial alterations promote further ROS release, creating an environment of oxidative injury [41]. In parallel, oxidative stress reduces NO bioavailability by promoting its degradation and impairing endothelial nitric oxide synthase activity, resulting in diminished vasodilation and increased vascular resistance, as explained above. Excess ROS also induces lipid peroxidation, generating reactive lipid species damaging cellular membranes and exacerbate endothelial dysfunction. In addition, oxidative stress activates multiple pro-inflammatory signaling pathways, which amplify the inflammatory state observed in PE [42].

Nonetheless, it is unlikely that a single factor causes endothelial dysfunction. It appears more likely to be attributed to some circulating factors previously reported to mediate this process in PE, such as total cell-free DNA (cfDNA), miRNA, placental growth factor (PlGF), soluble endoglin (sENG), soluble Fms-like tyrosine kinase 1 (sFlt-1), circulating syncytiotrophoblast-derived microparticles (STBMs), vascular endothelial growth factor (VEGF), and cluster of differentiation 93 (CD93) [43,44].

Endothelial cell activation is an essential part of the inflammatory response that is exacerbated in PE. Chronic immune activation with the release of pro-inflammatory mediators, such as cytokines, may play a role in mediating endothelial dysfunction during pregnancy [45,46,47,48,49]. Endothelial cells could recognize danger signals and regulate inflammatory response [50] through receptors that recognize a range of external signals [51].

Danger-associated signals interacting with endothelial cells could trigger activation of the NOD-like receptor pyrin domain–containing protein 3 (NLRP3) [15]. Moreover, accumulating evidence indicates that pregnant women with PE exhibit significantly increased expression of NLRP3 and its associated mediators, including caspase-1, interleukin 1 beta (IL-1β), and interleukin 18 (IL-18), when compared with normotensive healthy pregnant women [52,53]. Thus, modulation of endothelial inflammation or activation may be clinically relevant in PE, as inflammatory signaling in endothelial cells is closely linked to endothelial dysfunction, a central feature of the disease. In the following section, NLRP3 inflammasome activation and its regulatory mechanisms in PE will be discussed in detail.

## 4. NLRP3 Inflammasome Activation and Its Regulatory Mechanisms in Preeclampsia

The NLRP3 inflammasome is a key component of the innate immune system involved in host defense against microbial pathogens. Nevertheless, dysregulated activation of this complex has been implicated in several inherited autoinflammatory diseases and in a variety of inflammatory-sterile conditions, including PE. Importantly, controlled inflammasome activation is not inherently detrimental during pregnancy. Controlled innate immune responses are required for normal placental development and host defense, suggesting that pathological outcomes are more likely related to excessive, sustained, or improperly regulated activation of the inflammasome rather than to inflammasome signaling itself [12].

**Figure 1:**
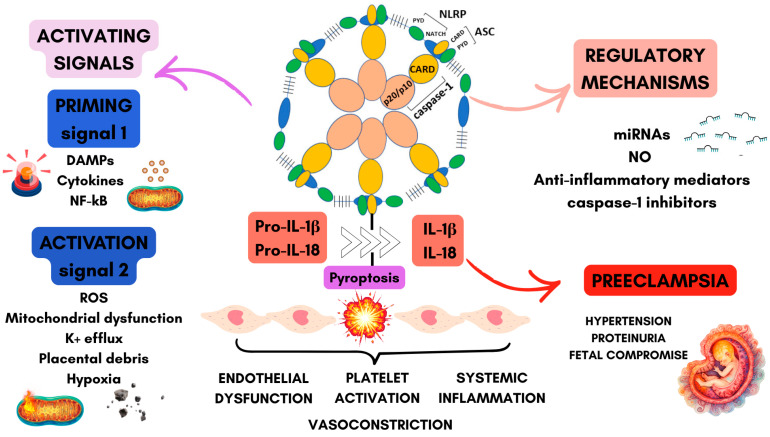
NLRP3 inflammasome activation and regulatory mechanisms in preeclampsia (PE). The NLRP3 activation occurs in two steps: priming and activation. Priming, mediated by PRRs and inflammatory cytokines, induces NF-κB–dependent upregulation of NLRP3 and pro–IL-1β. Activation is triggered by PE-associated cellular stress signals. These events promote assembly of the NLRP3 inflammasome complex (NLRP3, ASC, and pro–caspase-1—represented by the elements rose yellow—caspase-1, yellow green and blue—ASC, and green and blue—NLRP) leading to caspase-1 activation, IL-1β and IL-18 maturation, and gasdermin D–mediated pyroptosis. NLRP3 activity is regulated by mechanisms such as post-translational modifications, autophagy, nitric oxide signaling, and miRNA-mediated repression. Dysregulation of these pathways may contribute to inflammation and endothelial dysfunction in PE. Graphical elements were adapted from Servier Medical (https://smart.servier.com/, accessed between 1 December 2025 and 1 March 2026), licensed under CC BY 4.0 and created using Canva (https://www.canva.com/, accessed between 1 December 2025 and 1 March 2026).

Under basal conditions, NLRP3 remains in an inactive oligomeric conformation in the cytosol. In response to activating stimuli, it assembles into a multiprotein complex (as illustrated in Figure 1), leading to activation of caspase-1 and the subsequent maturation and secretion of the pro-inflammatory cytokines IL-1β and IL-18 [54]. Several cellular events have been implicated in NLRP3 activation, including potassium (K^+^) efflux, ROS generation, and lysosomal destabilization, with cathepsin B release. These signals often arise from disturbances in intracellular homeostasis, triggered by diverse stimuli such as DAMPs, inflammatory cytokines, and hypoxic conditions [55]. Despite significant advances, the precise molecular mechanisms driving NLRP3 activation remain incompletely characterized.

Activation of the NLRP3 inflammasome generally occurs through a two-step process consisting of a priming phase followed by an activation phase (Figure 1) [56]. During priming, inflammatory signaling pathways increase the transcription of inflammasome components and related cytokines, preparing the cell for subsequent activation. The second step involves the recognition of specific stimuli that promote inflammasome assembly and full activation. Multiple endogenous regulatory mechanisms, including autophagy, mitophagy, and tightly controlled post-translational modifications (PTMs), act to restrain excessive inflammasome activity under physiological conditions. PTMs such as phosphorylation, ubiquitination, and deacetylation play important roles in regulating NLRP3 stability, conformational state, and interactions with adaptor proteins during both the priming and activation phases [57,58].

Increasing evidence suggests that the NLRP3 inflammasome is involved in the pathophysiology of PE. Elevated expression of NLRP3 inflammasome components has been reported in placental tissues and peripheral blood mononuclear cells from women with PE compared with normotensive pregnancies [52,59,60]. In parallel, several DAMPs capable of activating the inflammasome—including high-mobility group box-1 (HMGB1), extracellular DNA, advanced glycation end products (AGEs), free fatty acids, cellular debris, and crystalline structures such as cholesterol and monosodium urate (MSU)—have been detected at increased levels in the circulation and placental tissues of patients with PE [61,62]. Nevertheless, the precise role of the NLRP3 inflammasome in the pathogenesis of PE remains debated. Inflammasome activation may occur as a downstream consequence of placental ischemia and the release of danger signals, acting as a potent amplifier of sterile inflammation once placental stress is established or potentially contributing to early pathogenic events that predispose to placental dysfunction [12,61,63]. These possibilities suggest that NLRP3 may participate in multiple stages of disease development, although the relative contribution of each mechanism remains to be fully elucidated.

Given that excessive inflammasome activation is associated with increased pro-inflammatory cytokine production and inflammatory cell death pathways such as pyroptosis, several endogenous mechanisms act to limit NLRP3 activity. These regulatory pathways include microRNAs, nitric oxide (NO), and anti-inflammatory mediators, which collectively contribute to maintaining immune balance during pregnancy. Disruption of these regulatory processes may promote excessive inflammation and contribute to pregnancy complications. From a therapeutic perspective, pharmacological inhibitors targeting the NLRP3 inflammasome have been proposed as potential strategies for the treatment of inflammasome-related diseases [64]. However, the development of such interventions for use during pregnancy requires careful consideration of safety, tissue specificity, and the potential consequences of broadly suppressing innate immune responses.

## 5. MiRNAs and Their Role in NLRP3 Inflammasome Regulation

MiRNAs are approximately 19–24 nucleotides in length, exert post-transcriptional effects, and are the most extensively studied non-coding RNAs [65]. Most miRNAs are generated through a pathway involving two RNase III enzymes. Primary miRNAs (pri-miRNAs) are cleaved by a nuclear microprocessor complex composed of the enzyme Drosha and the protein DGCR8. After the formation of the precursor miRNA (pre-miRNA), it is protected and transported to the cytoplasm, where it is processed by the Dicer/TRBP/PACT enzymatic complex, losing its hairpin structure and giving rise to two single-stranded miRNA molecules. One of these strands is associated with an Argonaute (Ago) protein, while the other is degraded [66].

The classical mechanism of action of miRNAs involves binding to the 3′ untranslated region (3′ UTR) of target mRNAs, leading to their degradation or translational repression [66]. This process requires the association of the miRNA with an Ago protein, which is the main component of the RNA-induced silencing complex (RISC). Once bound to an Ago protein, the miRNA can guide RISC to a complementary mRNA, resulting in translational repression or mRNA degradation. MiRNAs are also capable of inhibiting protein expression by binding to coding regions or to the 5′ untranslated region (5′ UTR) of mRNAs [67].

MiRNAs were recently shown to modulate inflammasome activity at the post-transcriptional level by binding to target mRNAs, thereby either suppressing or enhancing the expression of inflammasome-related genes [68]. However, different miRNAs have been reported to regulate distinct inflammasomes [69,70]. Among these, NLRP3 is the most extensively investigated inflammasome, and miR-223 is the most extensively characterized and consistently validated regulator of NLRP3 expression, acting as a negative feedback controller of inflammasome activation in myeloid and non-myeloid cells [71,72]. Additional miRNAs, including miR-7, miR-22, and members of the miR-30 family, have also been shown to directly suppress NLRP3 expression in various inflammatory and injury-related models [73,74,75]. Together, these findings support direct miRNA-mediated repression of NLRP3 as a conserved anti-inflammatory checkpoint.

Beyond direct targeting of NLRP3, miRNAs exert indirect control over inflammasome activity by modulating upstream priming pathways and downstream effector components. Several miRNAs regulate NF-κB–dependent transcription, thereby influencing the expression of inflammasome components and pro–IL-1β [76,77]. Other miRNAs affect inflammasome assembly and signaling by targeting adaptor proteins, caspase-1, or cytokine maturation pathways, ultimately shaping the magnitude and duration of the inflammatory response [78,79,80]. Through these coordinated actions, miRNAs function as system-level regulators that integrate inflammatory stimuli with cellular responses.

Importantly, miRNA–NLRP3 regulatory interactions are highly context-dependent and vary across disease states. As summarized in Table 1, miRNAs implicated in NLRP3 regulation have been identified in a broad range of pathological conditions.

Evidence from multiple disease models indicates that miRNAs regulate the NLRP3 inflammasome through diverse mechanisms. In neurological disorders, circulating miR-124-3p was significantly downregulated in patients with basal ganglia intracerebral hemorrhage, and mechanistic studies in LPS-stimulated microglial cells demonstrated that it suppresses NLRP3 activation by targeting TRAF6 [81]. In traumatic brain injury models, miR-29a-5p protected blood–brain barrier integrity by modulating NLRP3 signaling [82], while miR-423-5p was shown to regulate microglial polarization and inflammasome activation in cerebral ischemia/reperfusion injury models [83,84]. Pharmacological evidence further indicates that isoliquiritin inhibits NLRP3-mediated pyroptosis through the miR-27a/SYK/NF-κB axis [85].

In cancer contexts, altered urinary miRNAs targeting NLR inflammasomes have been reported in bladder cancer patients [86], and miR-22 has been shown to sustain NLRP3 expression in *Helicobacter pylori*-associated gastric cancer models [87], while broader analyses highlight miRNA control of pyroptosis through inflammasome components [88]. Similarly, miRNAs such as miR-223 and miR-146a regulate NLRP3-driven inflammation in intestinal inflammation and diabetic nephropathy models [80,89,90].

Several miRNAs also regulate the inflammasome in autoimmune and inflammatory diseases; experimental evidence from cellular and animal models shows that miR-20a, miR-26b, and miR-30a modulate NLRP3 activation in rheumatoid arthritis, while miR-223 and miR-369-3p regulate inflammasome-driven inflammation in autoimmune hepatitis and intestinal inflammation, respectively [89,91,92,93,94,95].

In CVD models, miRNAs including miR-30c-5p, miR-9, miR-1929-3p, miR-135b, and miR-703 modulate NLRP3-mediated cardiac inflammation and fibrosis in hypertension and virus-induced cardiac injury models, whereas miR-30a-5p has been associated with enhanced NLRP3 signaling [96,97,98,99,100,101,102,103,104,105]. Because endothelial dysfunction, oxidative stress, and inflammation are shared features of CVD and PE, these findings provide important mechanistic insights into how miRNA-mediated regulation of NLRP3 may contribute to the vascular and inflammatory alterations observed in preeclampsia.

Despite their diversity, many of these conditions share common upstream drivers of NLRP3 activation, such as oxidative stress, hypoxia, mitochondrial dysfunction, and the accumulation of DAMPs, which likely explain the recurrent involvement of specific miRNAs across distinct pathological contexts.

**Table 1:** MiRNAs implicated in direct and indirect regulation of the NLRP3 inflammasome across disease contexts.

Condition/Disease	miRNA	Biological Sample/Model	Implication	Author (Ref.)
Neurological disorders and central nervous system injuries	miR-124-3p	Patients/Serum and In vitro model (HMC3 cells)	Downregulated; Consequent upregulation of NLRP3	[81]
	miR-214	In vitro model (U87 and U251 cells)	Downregulated; Upregulation could suppress NLRP3	[106]
	miR-29a-3p and miR-29a-5p	In vitro model (bEnd.3 cells)	Downregulated; Upregulation could suppress NLRP3	[82]
	miR-30e	Animal model (C57BL/6 mice)	Reduces NLRP3 expression	[107]
	miR-7 and miR-190	Animal model (Mice)	Upregulation can reduce NLRP3 expression	
	miR-135b	In vitro model (SH-SY5Y and PC-12 cell)	Reduces NLRP3 expression	
	miR-223 and miR-152	Animal model and In vitro model (C57BL/6 mice and BV2 and HT22 cells)	Repressed NLRP3	
	miR-20b	Animal model and In vitro model (Sprague-Dawley rats and HUVEC cells)	Downregulated; Suppress NLRP3	
	miR-423-5p	Animal model and In vitro model (Sprague–Dawley rats and HAPI cells)	Downregulated; Upregulation could suppress NLRP3	[83,84]
	miR-27a	Serum; patients and animal model (Sprague-Dawley rats)	Downregulated; Upregulation could suppress NLRP3	[85]
Bladder cancer	miR-223-3p	Patients	Upregulated and target NLRP3	[86]
Gastric cancer	miR-22	Patients and In vitro model (AGS, THP-1, BGC-823, GES-1, SGC-7901, HGC-27 and BGC-823 cells)	Downregulated; NLRP3 is upregulated	[87]
Cancer	miR-21-5p	Patients and In vitro model (HCT-116 and HT-29 cells)	Could upregulate NLRP3	[88]
	miR-214, miR-181 and miR-200b	In vitro model (Hela, HCC94, Siha or HUCEL normal cells; sh-Sy5y cells; (MCF-7 and BT-549 cells)	Facilitate the assembly of NLRP3	
	miR-556-5p and miR-195	In vitro model (sh-Sy5y, A549 and H1299 cells)	Downregulates NLRP3	
Metabolic syndromes	miR-223	In vitro model (Primary monocytes)	Overexpression reduces the expression of NLRP3	[80,89]
	miR-20a	In vitro and In vivo model (Sprague-Dawley and Fibroblast-Like Synoviocyte cells)	Negatively regulates the expression of NLRP3	[80]
	miR-9	In vitro model (Immortalized human cardiomyocytes)	Involved in suppression of the NLRP3	
	miR-133	In vitro and In vivo model (THP1 cells and mice)	negative regulator of NLRP3 expression	
	miR-146a	In vivo and In vitro model (C57BL/6 mice and Thioglycollate-elicited peritoneal macrophages)	Downregulation increases NLRP3 expression	[90]
Infectious diseases	miR-223	Animal model (C57BL/6 mice)	Downregulated; Upregulation could suppress NLRP3	[108]
Atherosclerosis	miR-30c-5p	In vitro model (HAECs cells)	Impaired expression; Upregulation could suppress NLRP3	[96]
	miR-22	In vitro model (EA.hy926 human endothelial cells)	Downregulation could affect NLRP3 expression	[97]
	miR-9	In vitro model (human THP-1 derived macrophages)	Inhibited NLRP3	[98]
	MiR-296	In vitro model (LCL-2 and LCL-FD cells)	Increased; Represses IKBKE and enhances NLRP3	[99]
Myocardial infarction	miR-135b	In vitro and In vivo model (Neonatal mice ventricular cardiomyocytes and C57BL/6 mice)	Downregulated; Upregulation could suppress NLRP3	[109]
	miR-703	In vitro model (mouse cardiomyocytes)	Downregulated; Targets and represses NLRP3	[101]
Diabetic cardiomyopathy	miR-223	Animal model and In vitro model (Sprague-Dawley rats and H9c2 cardiac cells)	Highly expressed; Inhibition would suppress NLRP3	[102]
Chronic heart failure	miR-30a-5p	Animal model (Sprague Dawley rats)	Upregulated; Regulated NLRP3 pathway	[103]
Rheumatoid arthritis	miR-20a	Animal model and In vitro model (miR-223^-/y^ mice on a C57BL/6J and Sprague-Dawley rats/Fibroblast-Like Synoviocyte)	Over-expression decrease formation of NLRP3	[91,92]
	miR-26b	Animal model (Sprague-Dawley rats)	Downregulated; Upregulation could suppress NLRP3	[92]
	miR-30a	Animal model (NLRP3-deficient NLRP3^KO^ mice generated in a C57BL/6J)	Negatively mediates NLRP3 expression	[93]
Inflammatory bowel disease	miR-223	Animal model (miR-223^-/y^ mice on a C57BL/6J background)	Downregulated; Upregulation could suppress NLRP3	[89]
Autoimmune hepatitis		In vitro and In vivo model (C57BL/6 mice and bone marrow-derived mesenchymal stem cells—BMSCs)	Upregulated with downregulation of NLRP3	[94]
Inflammatory bowel disease	miR-369-3p	In vitro model (Mouse bone marrow-derived macrophages—BMDM)	Upregulation could suppress NLRP3	[95]
Endothelial dysfunction	miR-30c-5p	In vitro model (HAECs)	Might play a role inhibiting NLRP3	[96]
Hypertension	miR-135a-5p	Animal model (C57BL/6 mice)	Over-expression could inhibit NLRP3	[104]
	miR-1929-3p	Animal model (C57BL/6 mice)	Downregulated; Associated with activation of NLRP3	[105]
Preeclampsia	miR-124-3p	Placenta and In vitro model (HTR-8/SVneo cells)	Upregulated; positively correlated with NLRP3	[110]
	miR-520c-3p	Placenta	Downregulated; Upregulation could potentially reduce NLRP3	[111]
	miR-223-3p	Placenta and In vitro model (HTR-8/SVneo cells)	Downregulated; Upregulation could potentially reduce NLRP3	[112]
	miR-494	Placenta	Upregulated; Indirectly upregulates NLRP3	[113]
	miR-135	Placenta and blood	Downregulated; Upregulation could potentially reduce NLRP3	[114]
	miR-141-3p	In vitro model (HTR-8/SVneo cells)	Upregulated; Indirectly upregulates NLRP3	[115]

Collectively, the existing literature supports a model in which miRNAs function as multilayered regulators of NLRP3 inflammasome priming, activation, and resolution. However, most of the evidence underlying these regulatory interactions derives from non-pregnant disease models, which limit direct extrapolation to pregnancy-specific conditions. Therefore, how miRNA-mediated control of NLRP3 inflammasome operates within placental cell populations and at the maternal–fetal immune interface remains insufficiently characterized. In this context, integrating insights from established miRNA–NLRP3 regulatory networks provides a critical framework for understanding how similar mechanisms may contribute to the pathophysiology of PE.

## 6. MiRNA-Mediated Regulation of the NLRP3 Inflammasome in the Pathophysiology of Preeclampsia

NLRP3 inflammasome is highly implicated in the pathogenesis of atherosclerosis, myocardial injury, heart failure, and other CVDs through its capacity to drive pro-inflammatory cytokine release and pyroptotic cell death, linking innate immune signaling to vascular and myocardial dysfunction [116]. Elevated NLRP3 activity correlates with disease severity and prognosis in conditions such as acute coronary syndrome, and its activation in vascular cells, including endothelial cells, contributes to vascular inflammation, plaque development, and dysfunction [117,118].

Growing evidence underscores a shared inflammatory axis between PE and CVDs [119], with the NLRP3 inflammasome emerging as a central regulatory node in both conditions. In this context, increased expression and activation of NLRP3 have been reported in placentas and decidua of pregnant women with PE [52,120]. Additionally, experimental models indicate that activation of the NLRP3 inflammasome contributes to the development of pregnancy-induced hypertension and key features of PE, supporting its role in the exaggerated inflammatory state that characterizes the disease [121]. Consistent with these findings, increased NLRP3 expression in peripheral monocytes has been proposed as a potential risk factor for early-onset PE in human cohorts, further reinforcing the link between dysregulation of the NLRP3 inflammatory pathway and hypertensive disorders of pregnancy [122].

Importantly, miRNAs have emerged as critical modulators of NLRP3 inflammasome signaling in cardiovascular contexts. Several miRNAs, including miR-125a-5p and miR-22, negatively regulate NLRP3 expression in experimental models of CVD. In endothelial cells, central to the pathophysiology of both PE and CVD, inflammasome activity regulated by miRNA appears particularly relevant [123,124]. Although direct evidence for miR-125a-5p regulating the NLRP3 inflammasome in models of PE is currently limited, previous studies in vascular inflammation provide strong mechanistic support for such a regulatory axis. miR-125a-5p expression is downregulated in human vascular smooth muscle cells exposed to oxidized low-density lipoprotein, whereas NLRP3 and associated inflammasome components are upregulated. Restoration of miR-125a-5p expression suppresses NLRP3, ASC (Apoptosis-associated Speck-like protein containing a CARD), caspase-1, and IL-1β levels by targeting upstream inflammatory mediators such as CCL4 (Chemokine Ligand 4), indicating negative regulation of inflammasome activation by miR-125a-5p [124]. In PE, clinical profiling studies have reported the upregulation of miR-125a-5p in placental tissues and have shown effects on trophoblast proliferation, migration, and angiogenesis. However, direct links between the miR-125a-5p and NLRP3 signaling in the placenta remain to be established [125].

**Figure 2:**
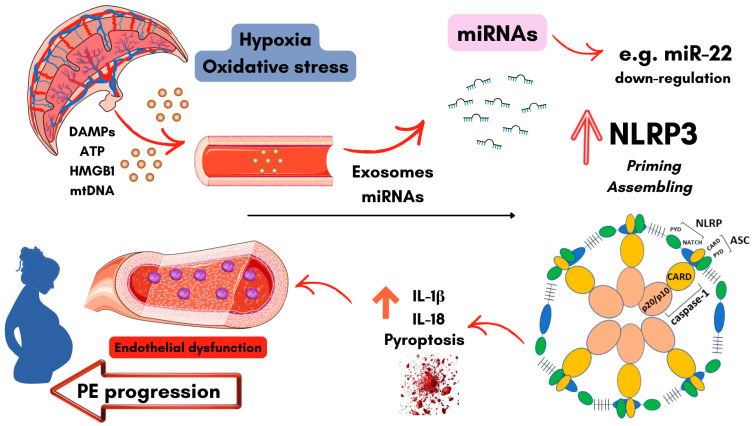
Example of possible miRNA-mediated regulation of the NLRP3 inflammasome in preeclampsia (PE). In the preeclamptic placenta, hypoxia, oxidative stress, and the release of DAMPs and extracellular vesicles containing dysregulated miRNAs may contribute to endothelial activation. Altered miRNA expression is capable of disrupting post-transcriptional regulation of inflammasome components and upstream signaling pathways, promoting NLRP3 priming and activation. This leads to caspase-1 activation, IL-1β and IL-18 release, endothelial inflammation, and endothelial dysfunction, contributing to PE progression. Graphical elements were adapted from Servier Medical (https://smart.servier.com/, accessed between 1 December 2025 and 1 March 2026), licensed under CC BY 4.0 and created using Canva (https://www.canva.com/, accessed between 1 December 2025 and 1 March 2026).

In the context of PE, placental hypoxia, oxidative stress, and increased release of damage-associated molecular patterns (DAMPs) drive exaggerated inflammatory signaling at the maternal–fetal interface, promoting endothelial activation and dysfunction. As illustrated in Figure 2, these stressors also alter placental and circulate miRNA expression profiles, disrupting post-transcriptional regulation of key inflammatory pathways. Placental tissues from women with PE consistently show increased expression of the NLRP3 inflammasome and associated components, supporting a pathogenic role for inflammasome activation [52]. Within this inflammatory environment, dysregulated miRNAs act as critical modulators of inflammasome priming and activation.

Importantly, several miRNAs have emerged as regulators of NLRP3 signaling in cardiovascular and vascular inflammation. For example, miR-125a-5p negatively regulates NLRP3 activation in experimental vascular models. In human vascular smooth muscle cells exposed to oxidized low-density lipoprotein, reduced miR-125a-5p expression is accompanied by increased levels of NLRP3, ASC, caspase-1, and IL-1β, while restoration of miR-125a-5p suppresses these inflammasome components by targeting upstream inflammatory mediators such as CCL4 [124]. Although direct evidence for miR-125a-5p regulating NLRP3 in placental cells remains limited, clinical studies have reported altered miR-125a-5p expression in placental tissues from women with PE and demonstrated effects on trophoblast proliferation, migration, and angiogenesis [125], suggesting a potential modulatory role in placental inflammation.

In parallel, miR-22 (as illustrated in Figure 2) has emerged as another negative regulator of NLRP3-driven inflammation. Experimental studies show that miR-22 directly targets NLRP3 and HIF-1α, suppressing caspase-1 activation and reducing IL-1β and IL-18 production under hypoxic and inflammatory conditions [123]. Placental and circulating miRNA profiling studies have also identified increased expression of miR-22-3p in placental tissue and maternal plasma from women who later develop PE, suggesting that its dysregulation may reflect early molecular alterations associated with PE pathophysiology [126].

Consistent with these observations, several studies have directly investigated miRNA–NLRP3 interactions in PE using placental samples and trophoblast models. Analyses of placental tissue from women with PE combined with functional experiments in HTR-8/SVneo trophoblast cells showed that miR-520c-3p and miR-223-3p suppress NLRP3 expression and downstream inflammatory signaling by directly targeting NLRP3 mRNA, with validation through RT-qPCR, Western blotting, and inflammasome-related assays [111,112]. In contrast, miR-124-3p promotes trophoblast pyroptosis in HTR-8/SVneo cells by targeting PLGF, suggesting a role in placental inflammatory injury [110]. Additional studies demonstrated that miR-135 attenuates inflammatory responses by restricting NLRP3 inflammasome activation through regulation of PCSK6 [114], whereas miR-141-3p expression is epigenetically regulated through promoter methylation and influences inflammasome formation and trophoblast invasiveness in trophoblast models [115]. Furthermore, miR-494 induces trophoblast senescence through targeting SIRT1, suggesting indirect effects on placental dysfunction [113]. Together, these findings support a role for miRNA-mediated regulation of inflammasome signaling in PE; however, most evidence relies on placental samples and in vitro trophoblast models, highlighting the need for further validation in larger clinical cohorts and in vivo systems.

Together, these findings support the model depicted in Figure 2, in which dysregulated miRNA-mediated control of inflammasome priming and activation amplifies placental and endothelial inflammation in PE. Such mechanisms may also contribute to the shared inflammatory axis linking PE to long-term maternal cardiovascular risk [112,127,128].

Taken together, the evidence reviewed here underscores that the pathophysiological impact of the NLRP3 inflammasome in pregnancy depends less on its absolute activation and more on the integrity of the regulatory networks that control its activity. Among these regulators, miRNAs emerge as key post-transcriptional checkpoints that fine-tune inflammasome priming and activation within placental and maternal vascular tissues. Disruption of these miRNA-mediated controls could increase the susceptibility of the placental and endothelial environment to exaggerated inflammatory responses triggered by gestational stressors such as hypoxia, oxidative stress, and DAMP release. In this context, early alterations in miRNA expression profiles could shift inflammatory set points well before the clinical onset of PE. Understanding how these miRNA–inflammasome regulatory layers evolve throughout pregnancy, and how they interact with maternal cardiovascular vulnerability, will be critical for clarifying why PE is associated with persistent vascular risk and for identifying potential windows for early detection and therapeutic intervention.

## 7. Limitations and Future Directions

Although accumulating evidence supports a role for NLRP3 inflammasome activation and miRNA-mediated regulation in PE, key mechanistic and translational gaps remain unknown. An important challenge is determining whether dysregulation of miRNA–NLRP3 networks represents a shared pathway across PE subtypes, such as early-onset and late-onset disease, term or preterm, or whether it preferentially contributes to distinct clinical phenotypes with divergent inflammatory and vascular profiles. Conversely, discrepancies reported in the literature data may partially reflect heterogeneity in experimental design, biological samples, gestational age at sampling, and analytical pipelines employed for miRNA profiling, underscoring the need for standardized methodologies.

Future studies should focus on identifying and functionally validating miRNAs that directly regulate NLRP3 signaling in placental, endothelial, and immune cells at the maternal–fetal interface using cell-type-specific and longitudinal approaches. Such strategies are particularly important to clarify temporal dynamics and causal relationships during disease onset and progression. In parallel, integrative analyses incorporating multiple epigenetic layers, including the crosstalk among DNA methylation and non-coding RNAs, are needed to better capture the regulatory complexity of the NLRP3 inflammasome in PE.

An additional gap is the limited clinical stratification across the available studies, as most investigations do not distinguish between different PE phenotypes (e.g., early- versus late-onset or disease severity), which precludes a more phenotype-oriented analysis of the miRNA–NLRP3 axis.

The clinical utility of NLRP3-associated miRNAs as predictive or prognostic biomarkers warrants evaluation in well-characterized, phenotypically stratified cohorts, ideally combined with established inflammatory, angiogenic, and vascular markers. Finally, although still in its early stages, therapeutic targeting of miRNA–NLRP3 networks represents a promising but largely unexplored avenue that may support the development of more precise diagnostic and interventional strategies aimed at mitigating endothelial dysfunction and adverse maternal and fetal outcomes in PE.

## 8. Conclusions

PE is a complex multisystem disorder driven by placental dysfunction, immune imbalance, and endothelial injury, with inflammation representing one of the main central pathogenic axes. The evidence summarized in this review supports the NLRP3 inflammasome as an important mediator linking placental stress signals, innate immune activation, and endothelial dysfunction. Hypoxia and oxidative stress–related stimuli appear to be key triggers of NLRP3 activation, promoting inflammatory amplification and vascular dysregulation that contribute to the clinical manifestations of the disease.

Recent evidence further indicates that microRNAs act as relevant epigenetic regulators of the NLRP3 inflammasome. By targeting inflammasome components directly or modulating upstream inflammatory signaling pathways, miRNAs integrate environmental and cellular stress signals with inflammatory responses at the maternal–fetal interface and within the vascular endothelium. However, current evidence also highlights substantial heterogeneity in miRNA expression patterns in PE, reflecting differences in clinical and biological contexts. This variability underscores the complexity of NLRP3 regulation during pregnancy and highlights important gaps in current literature.

Integrating findings from both PE and CVD research suggests that miRNA-mediated regulation of the NLRP3 inflammasome may represent a mechanistic link between pregnancy-related endothelial dysfunction and long-term maternal cardiovascular risk. Nevertheless, the available evidence remains limited, and important questions remain regarding the temporal dynamics of miRNA expression, the functional validation of miRNA–NLRP3 interactions, and the influence of PE clinical heterogeneity on this regulatory axis. Future studies integrating molecular, clinical, and longitudinal approaches will be essential to clarify these mechanisms.

A deeper understanding of the miRNA–NLRP3 regulatory network may provide further insight into the molecular mechanisms regulating inflammasome activity in PE and may help identify potential biomarkers or therapeutic targets. These findings could contribute to future strategies aimed at improving risk assessment and mitigating adverse pregnancy outcomes and long-term cardiovascular risks associated with PE.

## Data Availability

No new data were created or analyzed in this study.
